# Bis[*N*-benzyl-2-(quinolin-8-yl­oxy)acetamide] monohydrate

**DOI:** 10.1107/S1600536811019817

**Published:** 2011-06-04

**Authors:** Ming-Shi Wang, Hai-Yan Li, Wei-Na Wu

**Affiliations:** a Institute of Resources & Environment, Henan Polytechnic University, Jiaozuo 454000, People’s Republic of China; b Department of Physics and Chemistry, Henan Polytechnic University, Jiaozuo 454000, People’s Republic of China

## Abstract

In the title compound, 2C_18_H_16_N_2_O_2_·H_2_O, the dihedral angles between the quinoline rings and the benzene rings in the two independent acetamide mol­ecules are 80.09 (5) and 61.23 (5)°. The crystal packing is stablized by O—H⋯N and N—H⋯O hydrogen bonds between the acetamide and water mol­ecules.

## Related literature

For the luminescent properties of lanthanide complexes with amide-type ligands, see: Li *et al.* (2003 ▶); Wu *et al.* (2006 ▶). For the synthesis of 2-chloro-*N*-benzyl­acetamide and *N*-benzyl-2-(quinolin-8-yl­oxy)acetamide, see: Wu *et al.* (2006 ▶). For the structure of a copper(II) complex with *N*-benzyl-2-(quinolin-8-yl­oxy)acetamide, see: Wang *et al.* (2010 ▶).
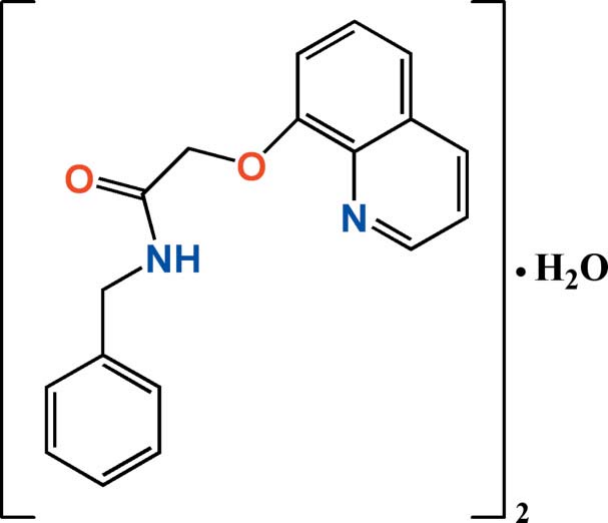


## Experimental

### 

#### Crystal data

2C_18_H_16_N_2_O_2_·H_2_O
*M* *_r_* = 602.67
Monoclinic, 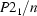

*a* = 13.7802 (12) Å
*b* = 12.3129 (11) Å
*c* = 18.9865 (17) Å
β = 101.066 (2)°
*V* = 3161.6 (5) Å^3^
*Z* = 4
Mo *K*α radiation
μ = 0.09 mm^−1^
*T* = 296 K
0.21 × 0.16 × 0.15 mm

#### Data collection

Bruker APEXII CCD diffractometer
Absorption correction: multi-scan (*SADABS*; Bruker, 2007 ▶) *T*_min_ = 0.984, *T*_max_ = 0.987
16274 measured reflections
5562 independent reflections
3572 reflections with *I* > 2σ(*I*)
*R*_int_ = 0.036

#### Refinement

*R*[*F* ^2^ > 2σ(*F* ^2^)] = 0.040
*wR*(*F* ^2^) = 0.092
*S* = 1.10
5562 reflections
413 parameters
10 restraints
H atoms treated by a mixture of independent and constrained refinement
Δρ_max_ = 0.15 e Å^−3^
Δρ_min_ = −0.13 e Å^−3^

### 

Data collection: *APEX2* (Bruker, 2007 ▶); cell refinement: *SAINT* (Bruker, 2007 ▶); data reduction: *SAINT*; program(s) used to solve structure: *SHELXS97* (Sheldrick, 2008 ▶); program(s) used to refine structure: *SHELXL97* (Sheldrick, 2008 ▶); molecular graphics: *SHELXTL* (Sheldrick, 2008 ▶); software used to prepare material for publication: *SHELXTL*.

## Supplementary Material

Crystal structure: contains datablock(s) I, global. DOI: 10.1107/S1600536811019817/vm2098sup1.cif

Structure factors: contains datablock(s) I. DOI: 10.1107/S1600536811019817/vm2098Isup2.hkl

Supplementary material file. DOI: 10.1107/S1600536811019817/vm2098Isup3.cml

Additional supplementary materials:  crystallographic information; 3D view; checkCIF report

## Figures and Tables

**Table 1:** Hydrogen-bond geometry (Å, °)

*D*—H⋯*A*	*D*—H	H⋯*A*	*D*⋯*A*	*D*—H⋯*A*
N2—H2*A*⋯O5^i^	0.86	2.09	2.903 (2)	157
N4—H4*A*⋯O5	0.86	2.10	2.9015 (19)	154
O5—H5*B*⋯N1^ii^	0.88 (1)	2.01 (2)	2.869 (2)	167 (2)
O5—H5*C*⋯N3	0.88 (1)	1.91 (2)	2.7849 (19)	173 (2)

Symmetry codes: (i) 
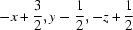
; (ii) 
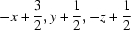
.

**Fig. 1.**
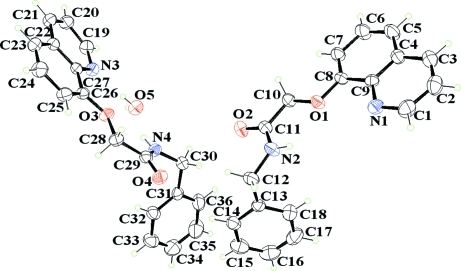
The title compound with the displacement ellipsoids shown at the 30% probability level.

**Fig. 2.**
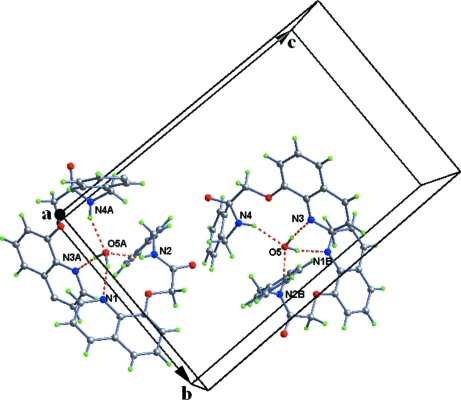
Part of the crystal packing for the title compound (hydrogen bonds shown as dashed lines, symmetry code: A: 1.5 - x, -1/2 + y, 0.5 - z; B: 1.5 - x, 1/2 + y, 0.5 - z).

## supplementary crystallographic information

### Comment

The amide type open-chain ligands have attracted much attention mainly because of their excellent coordination ability and high selectivity to metal ions (Li *et al.*, 2003 & Wu *et al.*, 2006). Previously, we have reported the structure of the copper(II) complex with the title acetamide molecular (Wang *et al.*, 2010). In this paper, the title compound was synthesized and characterized by X-ray diffraction.

In the title compound, 2C_18_H_16_N_2_O_2_.H_2_O, there are two independent *N*-benzyl-2-(quinolin-8-yloxy)acetamide molecules and a water molecule in the asymmetric unit (Fig. 1). Bond lengths and angles of the acetamide molecular are comparable with those observed in its copper(II) complex (Wang *et al.*, 2010). The dihedral angles between the quinoline rings (N1/C1–C9, r.m.s. deviation 0.0092Å and N3/C19–C27, r.m.s. deviation 0.0293 Å) and the benzene rings (C13–C18, r.m.s. deviation 0.0028Å and C31–C35, r.m.s. deviation 0.0039 Å) in two independent acetamide molecules are 80.09 (5)° and 61.23 (5)°, respectively. In the crystal structure, solvent water molecules form intermolecular O—H···N and N—H···O hydrogen bonds with acetamide molecules to stabilize the packing (Table 1, Fig. 2).

### Experimental

8-Hydroxyquinoline (1.5 g, 10.3 mmol) and anhydrous potassium carbonate (1.6 g, 11.6 mmol) were added to DMF (15 mL), then 2-chloro-*N*-benzylacetamide (1.83 g, 10.0 mmol) and a small quantity of KI were added. The reaction mixture was stirred for 5 h at 100–110 °C. After cooling down, 150 mL water was added and stirred for 2 h. The precipitate was collected by filtration and washed with water. Recrystallization from EtOH/H_2_O (1:1) gave colorless blocks.

### Refinement

The water H atoms were located from difference Fourier map calculation and then refined with O—H = 0.87Å and *U*_iso_(H) = 1.5*U*_eq_(O). Other H atoms attached to C and N atoms were placed in calculated positions and treated with the carrier atom-H distances = 0.93 Å for aryl, 0.97 for methylene, and 0.86 Å for the secondary amine H atoms. The *U*_iso_ values were constrained to be 1.2*U*_eq_ of the carrier atom for the H atoms.

### Crystal data

**Table:**

2C_18_H_16_N_2_O_2_·H_2_O	*F*(000) = 1272
*M**_r_* = 602.67	*D*_x_ = 1.266 Mg m^−^^3^
Monoclinic, *P*2_1_/*n*	Mo *K*α radiation, λ = 0.71073 Å
Hall symbol: -P 2yn	Cell parameters from 2885 reflections
*a* = 13.7802 (12) Å	θ = 2.4–21.3°
*b* = 12.3129 (11) Å	µ = 0.09 mm^−^^1^
*c* = 18.9865 (17) Å	*T* = 296 K
β = 101.066 (2)°	Colorless, block
*V* = 3161.6 (5) Å^3^	0.21 × 0.16 × 0.15 mm
*Z* = 4	

### Data collection

**Table:**

Bruker APEXII CCD diffractometer	5562 independent reflections
Radiation source: fine-focus sealed tube	3572 reflections with *I* > 2σ(*I*)
graphite	*R*_int_ = 0.036
φ and ω scans	θ_max_ = 25.0°, θ_min_ = 1.7°
Absorption correction: multi-scan (*SADABS*; Bruker, 2007)	*h* = −16→7
*T*_min_ = 0.984, *T*_max_ = 0.987	*k* = −14→14
16274 measured reflections	*l* = −22→22

### Refinement

**Table:**

Refinement on *F*^2^	Secondary atom site location: difference Fourier map
Least-squares matrix: full	Hydrogen site location: inferred from neighbouring sites
*R*[*F*^2^ > 2σ(*F*^2^)] = 0.040	H atoms treated by a mixture of independent and constrained refinement
*wR*(*F*^2^) = 0.092	*w* = 1/[σ^2^(*F*_o_^2^) + (0.0263*P*)^2^ + 0.250*P*] where *P* = (*F*_o_^2^ + 2*F*_c_^2^)/3
*S* = 1.10	(Δ/σ)_max_ < 0.001
5562 reflections	Δρ_max_ = 0.15 e Å^−^^3^
413 parameters	Δρ_min_ = −0.13 e Å^−^^3^
10 restraints	Extinction correction: *SHELXL97* (Sheldrick, 2008), Fc^*^=kFc[1+0.001xFc^2^λ^3^/sin(2θ)]^-1/4^
Primary atom site location: structure-invariant direct methods	Extinction coefficient: 0.0044 (3)

### Special details

**Table:**

Geometry. All e.s.d.'s (except the e.s.d. in the dihedral angle between two l.s. planes) are estimated using the full covariance matrix. The cell e.s.d.'s are taken into account individually in the estimation of e.s.d.'s in distances, angles and torsion angles; correlations between e.s.d.'s in cell parameters are only used when they are defined by crystal symmetry. An approximate (isotropic) treatment of cell e.s.d.'s is used for estimating e.s.d.'s involving l.s. planes.
Refinement. Refinement of *F*^2^ against ALL reflections. The weighted *R*-factor *wR* and goodness of fit *S* are based on *F*^2^, conventional *R*-factors *R* are based on *F*, with *F* set to zero for negative *F*^2^. The threshold expression of *F*^2^ > σ(*F*^2^) is used only for calculating *R*-factors(gt) *etc*. and is not relevant to the choice of reflections for refinement. *R*-factors based on *F*^2^ are statistically about twice as large as those based on *F*, and *R*- factors based on ALL data will be even larger.

### Fractional atomic coordinates and isotropic or equivalent isotropic displacement parameters (Å^2^)

**Table:**

	*x*	*y*	*z*	*U*_iso_*/*U*_eq_	
C1	0.87370 (17)	0.35339 (17)	−0.11790 (12)	0.0787 (6)	
H1B	0.8560	0.2805	−0.1226	0.094*	
C2	0.91662 (17)	0.3997 (2)	−0.17127 (12)	0.0799 (6)	
H2B	0.9271	0.3585	−0.2102	0.096*	
C3	0.94260 (14)	0.50466 (19)	−0.16585 (11)	0.0698 (6)	
H3B	0.9714	0.5370	−0.2011	0.084*	
C4	0.92611 (13)	0.56563 (16)	−0.10673 (10)	0.0556 (5)	
C5	0.95096 (16)	0.67581 (17)	−0.09803 (12)	0.0781 (6)	
H5A	0.9810	0.7108	−0.1316	0.094*	
C6	0.93149 (17)	0.73109 (17)	−0.04118 (12)	0.0791 (7)	
H6A	0.9477	0.8044	−0.0362	0.095*	
C7	0.88731 (14)	0.68027 (15)	0.01061 (10)	0.0602 (5)	
H7A	0.8741	0.7200	0.0494	0.072*	
C8	0.86372 (12)	0.57322 (13)	0.00444 (9)	0.0464 (4)	
C9	0.88205 (12)	0.51216 (14)	−0.05517 (9)	0.0469 (4)	
C10	0.79939 (13)	0.57628 (14)	0.11195 (9)	0.0531 (5)	
H10A	0.7561	0.6366	0.0947	0.064*	
H10B	0.8604	0.6057	0.1393	0.064*	
C11	0.75101 (12)	0.50584 (15)	0.15982 (10)	0.0507 (5)	
C12	0.68556 (13)	0.32963 (15)	0.18443 (10)	0.0628 (5)	
H12A	0.7049	0.3466	0.2351	0.075*	
H12B	0.7050	0.2552	0.1777	0.075*	
C13	0.57463 (13)	0.33827 (13)	0.16305 (10)	0.0501 (5)	
C14	0.51693 (15)	0.33263 (15)	0.21406 (11)	0.0618 (5)	
H14A	0.5468	0.3241	0.2620	0.074*	
C15	0.41588 (18)	0.33931 (18)	0.19589 (15)	0.0835 (7)	
H15A	0.3781	0.3365	0.2315	0.100*	
C16	0.37050 (18)	0.35013 (18)	0.12556 (18)	0.0915 (8)	
H16A	0.3019	0.3536	0.1131	0.110*	
C17	0.4266 (2)	0.35577 (18)	0.07423 (14)	0.0891 (7)	
H17A	0.3962	0.3637	0.0263	0.107*	
C18	0.52845 (18)	0.34986 (16)	0.09251 (11)	0.0748 (6)	
H18A	0.5661	0.3537	0.0568	0.090*	
C19	0.91850 (15)	0.82935 (15)	0.58711 (10)	0.0615 (5)	
H19A	0.8792	0.8888	0.5702	0.074*	
C20	1.00696 (16)	0.84872 (16)	0.63498 (10)	0.0664 (6)	
H20A	1.0270	0.9192	0.6480	0.080*	
C21	1.06269 (14)	0.76299 (17)	0.66198 (10)	0.0616 (5)	
H21A	1.1219	0.7741	0.6941	0.074*	
C22	1.03174 (13)	0.65699 (14)	0.64190 (9)	0.0489 (4)	
C23	1.08454 (14)	0.56364 (17)	0.66969 (10)	0.0621 (5)	
H23A	1.1423	0.5705	0.7040	0.075*	
C24	1.05166 (14)	0.46481 (16)	0.64678 (11)	0.0666 (6)	
H24A	1.0867	0.4037	0.6661	0.080*	
C25	0.96578 (13)	0.45142 (15)	0.59436 (10)	0.0589 (5)	
H25A	0.9448	0.3821	0.5791	0.071*	
C26	0.91309 (12)	0.53972 (13)	0.56575 (9)	0.0455 (4)	
C27	0.94373 (12)	0.64563 (13)	0.59055 (8)	0.0426 (4)	
C28	0.80035 (13)	0.43302 (13)	0.48266 (9)	0.0532 (5)	
H28A	0.7779	0.3878	0.5182	0.064*	
H28B	0.8567	0.3976	0.4688	0.064*	
C29	0.71875 (13)	0.44405 (15)	0.41805 (9)	0.0505 (5)	
C30	0.60228 (13)	0.56233 (15)	0.34132 (9)	0.0553 (5)	
H30A	0.6172	0.5192	0.3020	0.066*	
H30B	0.6068	0.6382	0.3285	0.066*	
C31	0.49780 (13)	0.53916 (14)	0.34841 (9)	0.0521 (5)	
C32	0.47299 (15)	0.47357 (15)	0.40065 (10)	0.0630 (5)	
H32A	0.5229	0.4432	0.4349	0.076*	
C33	0.37549 (19)	0.45171 (19)	0.40346 (13)	0.0830 (7)	
H33A	0.3601	0.4075	0.4395	0.100*	
C34	0.30206 (19)	0.4952 (3)	0.35327 (16)	0.1053 (9)	
H34A	0.2363	0.4800	0.3545	0.126*	
C35	0.32491 (18)	0.5613 (3)	0.30124 (14)	0.1105 (10)	
H35A	0.2747	0.5915	0.2672	0.133*	
C36	0.42192 (17)	0.5833 (2)	0.29898 (11)	0.0799 (7)	
H36A	0.4366	0.6289	0.2634	0.096*	
N1	0.85643 (11)	0.40589 (12)	−0.06099 (8)	0.0617 (4)	
N2	0.73869 (11)	0.40108 (12)	0.14402 (8)	0.0618 (4)	
H2A	0.7627	0.3748	0.1090	0.074*	
N3	0.88705 (10)	0.73225 (11)	0.56424 (7)	0.0520 (4)	
N4	0.67708 (10)	0.54074 (11)	0.40453 (7)	0.0522 (4)	
H4A	0.6951	0.5927	0.4344	0.063*	
O1	0.82035 (9)	0.51697 (9)	0.05242 (6)	0.0553 (3)	
O2	0.72574 (9)	0.54811 (10)	0.21194 (7)	0.0646 (4)	
O3	0.82985 (8)	0.53630 (8)	0.51330 (6)	0.0529 (3)	
O4	0.69461 (9)	0.36261 (10)	0.38143 (6)	0.0639 (4)	
O5	0.71153 (10)	0.75704 (10)	0.46479 (7)	0.0636 (4)	
H5B	0.6847 (14)	0.8068 (15)	0.4879 (10)	0.095*	
H5C	0.7673 (12)	0.7433 (16)	0.4947 (10)	0.095*	

### Atomic displacement parameters (Å^2^)

**Table:**

	*U*^11^	*U*^22^	*U*^33^	*U*^12^	*U*^13^	*U*^23^
C1	0.0992 (18)	0.0567 (13)	0.0810 (16)	−0.0044 (12)	0.0190 (14)	−0.0165 (12)
C2	0.0878 (17)	0.0883 (18)	0.0662 (14)	0.0106 (14)	0.0211 (13)	−0.0160 (13)
C3	0.0696 (14)	0.0804 (15)	0.0626 (13)	0.0052 (12)	0.0205 (11)	0.0041 (11)
C4	0.0543 (12)	0.0619 (12)	0.0525 (11)	0.0013 (9)	0.0146 (9)	0.0029 (9)
C5	0.0982 (17)	0.0677 (14)	0.0760 (15)	−0.0174 (12)	0.0361 (13)	0.0100 (12)
C6	0.1123 (19)	0.0511 (12)	0.0811 (15)	−0.0213 (12)	0.0364 (14)	0.0007 (11)
C7	0.0753 (14)	0.0479 (11)	0.0616 (12)	−0.0082 (10)	0.0233 (11)	−0.0011 (9)
C8	0.0450 (10)	0.0435 (10)	0.0520 (11)	−0.0021 (8)	0.0126 (9)	0.0067 (9)
C9	0.0407 (10)	0.0473 (10)	0.0519 (11)	0.0005 (8)	0.0066 (8)	0.0016 (9)
C10	0.0550 (12)	0.0507 (11)	0.0568 (11)	0.0011 (9)	0.0189 (10)	0.0008 (9)
C11	0.0420 (11)	0.0552 (12)	0.0551 (12)	0.0071 (9)	0.0104 (9)	0.0093 (10)
C12	0.0583 (13)	0.0586 (12)	0.0729 (13)	−0.0076 (10)	0.0159 (11)	0.0134 (10)
C13	0.0549 (12)	0.0397 (10)	0.0544 (12)	−0.0066 (8)	0.0076 (10)	0.0020 (8)
C14	0.0631 (14)	0.0586 (12)	0.0647 (13)	−0.0057 (10)	0.0147 (11)	0.0054 (10)
C15	0.0627 (16)	0.0830 (16)	0.110 (2)	0.0028 (12)	0.0284 (15)	0.0045 (14)
C16	0.0584 (16)	0.0737 (16)	0.134 (2)	0.0036 (12)	−0.0031 (18)	0.0037 (16)
C17	0.089 (2)	0.0841 (17)	0.0795 (18)	−0.0030 (15)	−0.0198 (16)	0.0007 (13)
C18	0.0819 (17)	0.0802 (15)	0.0610 (14)	−0.0047 (12)	0.0109 (12)	−0.0006 (11)
C19	0.0735 (15)	0.0461 (11)	0.0645 (12)	0.0022 (10)	0.0121 (11)	−0.0047 (10)
C20	0.0772 (15)	0.0544 (13)	0.0669 (13)	−0.0157 (11)	0.0123 (12)	−0.0133 (10)
C21	0.0568 (13)	0.0722 (14)	0.0538 (12)	−0.0127 (11)	0.0056 (10)	−0.0077 (10)
C22	0.0455 (11)	0.0563 (11)	0.0456 (10)	−0.0053 (9)	0.0108 (9)	−0.0006 (9)
C23	0.0495 (12)	0.0729 (14)	0.0600 (12)	−0.0027 (10)	0.0007 (10)	0.0103 (11)
C24	0.0558 (13)	0.0603 (13)	0.0794 (14)	0.0093 (10)	0.0021 (11)	0.0141 (11)
C25	0.0545 (12)	0.0469 (11)	0.0734 (13)	0.0010 (9)	0.0071 (10)	0.0034 (10)
C26	0.0397 (10)	0.0476 (11)	0.0491 (10)	−0.0011 (8)	0.0086 (9)	0.0004 (8)
C27	0.0428 (10)	0.0441 (10)	0.0430 (10)	0.0002 (8)	0.0135 (8)	0.0010 (8)
C28	0.0554 (12)	0.0431 (10)	0.0603 (12)	−0.0026 (9)	0.0091 (10)	−0.0073 (9)
C29	0.0538 (12)	0.0485 (11)	0.0512 (11)	−0.0078 (9)	0.0154 (9)	−0.0055 (9)
C30	0.0585 (12)	0.0578 (11)	0.0509 (11)	−0.0023 (9)	0.0135 (10)	0.0030 (9)
C31	0.0554 (12)	0.0586 (12)	0.0441 (10)	−0.0001 (9)	0.0139 (10)	−0.0082 (9)
C32	0.0665 (14)	0.0653 (13)	0.0622 (12)	−0.0043 (10)	0.0251 (11)	−0.0038 (10)
C33	0.0854 (18)	0.0910 (17)	0.0835 (17)	−0.0175 (14)	0.0439 (15)	−0.0149 (13)
C34	0.0627 (18)	0.165 (3)	0.096 (2)	−0.0199 (17)	0.0343 (16)	−0.035 (2)
C35	0.0592 (17)	0.193 (3)	0.0783 (18)	0.0145 (18)	0.0098 (14)	−0.0052 (19)
C36	0.0666 (16)	0.1183 (19)	0.0562 (13)	0.0097 (14)	0.0148 (12)	0.0074 (13)
N1	0.0702 (11)	0.0470 (9)	0.0685 (11)	−0.0041 (8)	0.0150 (9)	−0.0059 (8)
N2	0.0648 (11)	0.0565 (10)	0.0700 (10)	−0.0057 (8)	0.0275 (9)	0.0043 (8)
N3	0.0569 (10)	0.0424 (9)	0.0556 (9)	0.0026 (7)	0.0082 (8)	−0.0024 (7)
N4	0.0559 (10)	0.0467 (9)	0.0523 (9)	−0.0028 (7)	0.0064 (8)	−0.0056 (7)
O1	0.0655 (8)	0.0461 (7)	0.0601 (8)	−0.0063 (6)	0.0267 (7)	−0.0006 (6)
O2	0.0719 (9)	0.0674 (9)	0.0594 (8)	0.0122 (7)	0.0253 (7)	0.0095 (7)
O3	0.0496 (8)	0.0419 (7)	0.0631 (8)	0.0008 (5)	0.0009 (6)	−0.0079 (6)
O4	0.0771 (9)	0.0502 (8)	0.0626 (8)	−0.0097 (6)	0.0090 (7)	−0.0123 (6)
O5	0.0640 (10)	0.0484 (8)	0.0742 (10)	0.0110 (6)	0.0025 (7)	−0.0026 (7)

### Geometric parameters (Å, °)

**Table:**

C1—N1	1.319 (2)	C19—H19A	0.9300
C1—C2	1.390 (3)	C20—C21	1.348 (3)
C1—H1B	0.9300	C20—H20A	0.9300
C2—C3	1.339 (3)	C21—C22	1.403 (2)
C2—H2B	0.9300	C21—H21A	0.9300
C3—C4	1.405 (2)	C22—C23	1.408 (2)
C3—H3B	0.9300	C22—C27	1.410 (2)
C4—C5	1.401 (3)	C23—C24	1.341 (3)
C4—C9	1.410 (2)	C23—H23A	0.9300
C5—C6	1.346 (3)	C24—C25	1.402 (2)
C5—H5A	0.9300	C24—H24A	0.9300
C6—C7	1.400 (3)	C25—C26	1.361 (2)
C6—H6A	0.9300	C25—H25A	0.9300
C7—C8	1.357 (2)	C26—O3	1.3677 (18)
C7—H7A	0.9300	C26—C27	1.423 (2)
C8—O1	1.3696 (18)	C27—N3	1.359 (2)
C8—C9	1.421 (2)	C28—O3	1.4247 (18)
C9—N1	1.354 (2)	C28—C29	1.503 (2)
C10—O1	1.4210 (19)	C28—H28A	0.9700
C10—C11	1.502 (2)	C28—H28B	0.9700
C10—H10A	0.9700	C29—O4	1.2292 (19)
C10—H10B	0.9700	C29—N4	1.325 (2)
C11—O2	1.226 (2)	C30—N4	1.449 (2)
C11—N2	1.328 (2)	C30—C31	1.499 (2)
C12—N2	1.454 (2)	C30—H30A	0.9700
C12—C13	1.508 (2)	C30—H30B	0.9700
C12—H12A	0.9700	C31—C32	1.372 (2)
C12—H12B	0.9700	C31—C36	1.376 (2)
C13—C14	1.368 (2)	C32—C33	1.381 (3)
C13—C18	1.375 (3)	C32—H32A	0.9300
C14—C15	1.371 (3)	C33—C34	1.360 (3)
C14—H14A	0.9300	C33—H33A	0.9300
C15—C16	1.368 (3)	C34—C35	1.362 (3)
C15—H15A	0.9300	C34—H34A	0.9300
C16—C17	1.357 (3)	C35—C36	1.373 (3)
C16—H16A	0.9300	C35—H35A	0.9300
C17—C18	1.381 (3)	C36—H36A	0.9300
C17—H17A	0.9300	N2—H2A	0.8600
C18—H18A	0.9300	N4—H4A	0.8600
C19—N3	1.317 (2)	O5—H5B	0.875 (14)
C19—C20	1.394 (2)	O5—H5C	0.880 (14)
			
N1—C1—C2	124.4 (2)	C20—C21—C22	120.27 (18)
N1—C1—H1B	117.8	C20—C21—H21A	119.9
C2—C1—H1B	117.8	C22—C21—H21A	119.9
C3—C2—C1	119.0 (2)	C21—C22—C23	123.33 (17)
C3—C2—H2B	120.5	C21—C22—C27	117.17 (16)
C1—C2—H2B	120.5	C23—C22—C27	119.49 (16)
C2—C3—C4	119.7 (2)	C24—C23—C22	120.07 (18)
C2—C3—H3B	120.2	C24—C23—H23A	120.0
C4—C3—H3B	120.2	C22—C23—H23A	120.0
C3—C4—C5	122.57 (19)	C23—C24—C25	121.55 (18)
C3—C4—C9	117.55 (18)	C23—C24—H24A	119.2
C5—C4—C9	119.88 (18)	C25—C24—H24A	119.2
C6—C5—C4	120.15 (19)	C26—C25—C24	120.15 (17)
C6—C5—H5A	119.9	C26—C25—H25A	119.9
C4—C5—H5A	119.9	C24—C25—H25A	119.9
C5—C6—C7	121.20 (19)	C25—C26—O3	125.07 (16)
C5—C6—H6A	119.4	C25—C26—C27	119.88 (16)
C7—C6—H6A	119.4	O3—C26—C27	115.06 (14)
C8—C7—C6	120.18 (18)	N3—C27—C22	122.26 (15)
C8—C7—H7A	119.9	N3—C27—C26	118.97 (15)
C6—C7—H7A	119.9	C22—C27—C26	118.77 (15)
C7—C8—O1	124.16 (16)	O3—C28—C29	111.27 (14)
C7—C8—C9	120.41 (16)	O3—C28—H28A	109.4
O1—C8—C9	115.42 (15)	C29—C28—H28A	109.4
N1—C9—C4	122.33 (16)	O3—C28—H28B	109.4
N1—C9—C8	119.51 (16)	C29—C28—H28B	109.4
C4—C9—C8	118.16 (16)	H28A—C28—H28B	108.0
O1—C10—C11	111.48 (15)	O4—C29—N4	124.27 (17)
O1—C10—H10A	109.3	O4—C29—C28	117.83 (16)
C11—C10—H10A	109.3	N4—C29—C28	117.90 (15)
O1—C10—H10B	109.3	N4—C30—C31	115.70 (15)
C11—C10—H10B	109.3	N4—C30—H30A	108.4
H10A—C10—H10B	108.0	C31—C30—H30A	108.4
O2—C11—N2	123.42 (17)	N4—C30—H30B	108.4
O2—C11—C10	118.10 (17)	C31—C30—H30B	108.4
N2—C11—C10	118.47 (17)	H30A—C30—H30B	107.4
N2—C12—C13	113.72 (15)	C32—C31—C36	117.61 (18)
N2—C12—H12A	108.8	C32—C31—C30	123.63 (17)
C13—C12—H12A	108.8	C36—C31—C30	118.73 (17)
N2—C12—H12B	108.8	C31—C32—C33	121.5 (2)
C13—C12—H12B	108.8	C31—C32—H32A	119.3
H12A—C12—H12B	107.7	C33—C32—H32A	119.3
C14—C13—C18	118.14 (19)	C34—C33—C32	119.6 (2)
C14—C13—C12	120.18 (17)	C34—C33—H33A	120.2
C18—C13—C12	121.67 (19)	C32—C33—H33A	120.2
C13—C14—C15	121.3 (2)	C35—C34—C33	119.9 (2)
C13—C14—H14A	119.4	C35—C34—H34A	120.0
C15—C14—H14A	119.4	C33—C34—H34A	120.0
C16—C15—C14	120.2 (2)	C34—C35—C36	120.2 (2)
C16—C15—H15A	119.9	C34—C35—H35A	119.9
C14—C15—H15A	119.9	C36—C35—H35A	119.9
C17—C16—C15	119.3 (2)	C35—C36—C31	121.2 (2)
C17—C16—H16A	120.4	C35—C36—H36A	119.4
C15—C16—H16A	120.4	C31—C36—H36A	119.4
C16—C17—C18	120.5 (2)	C1—N1—C9	117.06 (17)
C16—C17—H17A	119.7	C11—N2—C12	121.60 (16)
C18—C17—H17A	119.7	C11—N2—H2A	119.2
C13—C18—C17	120.5 (2)	C12—N2—H2A	119.2
C13—C18—H18A	119.7	C19—N3—C27	117.51 (15)
C17—C18—H18A	119.7	C29—N4—C30	122.57 (15)
N3—C19—C20	124.17 (18)	C29—N4—H4A	118.7
N3—C19—H19A	117.9	C30—N4—H4A	118.7
C20—C19—H19A	117.9	C8—O1—C10	116.78 (13)
C21—C20—C19	118.50 (18)	C26—O3—C28	117.29 (12)
C21—C20—H20A	120.7	H5B—O5—H5C	102.2 (16)
C19—C20—H20A	120.8		
			
N1—C1—C2—C3	−0.2 (3)	C24—C25—C26—C27	−1.8 (3)
C1—C2—C3—C4	0.0 (3)	C21—C22—C27—N3	−3.9 (2)
C2—C3—C4—C5	−179.6 (2)	C23—C22—C27—N3	176.83 (16)
C2—C3—C4—C9	−0.1 (3)	C21—C22—C27—C26	176.37 (16)
C3—C4—C5—C6	178.5 (2)	C23—C22—C27—C26	−2.9 (2)
C9—C4—C5—C6	−1.0 (3)	C25—C26—C27—N3	−176.31 (16)
C4—C5—C6—C7	0.7 (3)	O3—C26—C27—N3	3.6 (2)
C5—C6—C7—C8	0.4 (3)	C25—C26—C27—C22	3.4 (2)
C6—C7—C8—O1	−179.94 (17)	O3—C26—C27—C22	−176.72 (13)
C6—C7—C8—C9	−1.1 (3)	O3—C28—C29—O4	171.37 (15)
C3—C4—C9—N1	0.4 (3)	O3—C28—C29—N4	−9.1 (2)
C5—C4—C9—N1	179.90 (17)	N4—C30—C31—C32	−18.7 (3)
C3—C4—C9—C8	−179.18 (15)	N4—C30—C31—C36	163.07 (17)
C5—C4—C9—C8	0.4 (3)	C36—C31—C32—C33	0.5 (3)
C7—C8—C9—N1	−178.87 (16)	C30—C31—C32—C33	−177.74 (17)
O1—C8—C9—N1	0.1 (2)	C31—C32—C33—C34	0.5 (3)
C7—C8—C9—C4	0.7 (2)	C32—C33—C34—C35	−1.0 (4)
O1—C8—C9—C4	179.66 (14)	C33—C34—C35—C36	0.5 (4)
O1—C10—C11—O2	−177.43 (14)	C34—C35—C36—C31	0.4 (4)
O1—C10—C11—N2	3.2 (2)	C32—C31—C36—C35	−0.9 (3)
N2—C12—C13—C14	−141.83 (17)	C30—C31—C36—C35	177.4 (2)
N2—C12—C13—C18	39.4 (2)	C2—C1—N1—C9	0.4 (3)
C18—C13—C14—C15	−0.6 (3)	C4—C9—N1—C1	−0.5 (3)
C12—C13—C14—C15	−179.42 (18)	C8—C9—N1—C1	179.00 (17)
C13—C14—C15—C16	1.0 (3)	O2—C11—N2—C12	5.6 (3)
C14—C15—C16—C17	−1.0 (3)	C10—C11—N2—C12	−175.07 (15)
C15—C16—C17—C18	0.5 (4)	C13—C12—N2—C11	79.7 (2)
C14—C13—C18—C17	0.1 (3)	C20—C19—N3—C27	1.6 (3)
C12—C13—C18—C17	178.91 (18)	C22—C27—N3—C19	1.7 (2)
C16—C17—C18—C13	−0.1 (3)	C26—C27—N3—C19	−178.58 (16)
N3—C19—C20—C21	−2.6 (3)	O4—C29—N4—C30	−4.5 (3)
C19—C20—C21—C22	0.1 (3)	C28—C29—N4—C30	175.96 (15)
C20—C21—C22—C23	−177.88 (18)	C31—C30—N4—C29	87.4 (2)
C20—C21—C22—C27	2.9 (3)	C7—C8—O1—C10	0.1 (2)
C21—C22—C23—C24	−178.48 (19)	C9—C8—O1—C10	−178.87 (14)
C27—C22—C23—C24	0.7 (3)	C11—C10—O1—C8	179.32 (13)
C22—C23—C24—C25	1.0 (3)	C25—C26—O3—C28	−4.9 (2)
C23—C24—C25—C26	−0.5 (3)	C27—C26—O3—C28	175.27 (14)
C24—C25—C26—O3	178.38 (16)	C29—C28—O3—C26	−170.51 (13)

### Hydrogen-bond geometry (Å, °)

**Table:**

*D*—H···*A*	*D*—H	H···*A*	*D*···*A*	*D*—H···*A*
N2—H2A···O5^i^	0.86	2.09	2.903 (2)	157
N4—H4A···O5	0.86	2.10	2.9015 (19)	154
O5—H5B···N1^ii^	0.88 (1)	2.01 (2)	2.869 (2)	167.(2)
O5—H5C···N3	0.88 (1)	1.91 (2)	2.7849 (19)	173.(2)

Symmetry codes: (i) −*x*+3/2, *y*−1/2, −*z*+1/2; (ii) −*x*+3/2, *y*+1/2, −*z*+1/2.

## References

[bb1] Bruker (2007). *APEX2*, *SAINT* and *SADABS* Bruker AXS Inc., Madison, Wisconsin, USA .

[bb2] Li, X.-F., Liu, W.-S., Guo, Z.-J. & Tan, M.-Y. (2003). *Inorg. Chem.* **42**, 8735–8738. 10.1021/ic034268d 14686851

[bb3] Sheldrick, G. M. (2008). *Acta Cryst.* A**64**, 112–122. 10.1107/S0108767307043930 18156677

[bb4] Wang, Y., Wu, W.-N., Zhao, R.-Q., Zhang, A.-Y. & Qin, B.-F. (2010). *Acta Cryst.* E**66**, m292. 10.1107/S1600536810005453 PMC2983527 21580237

[bb5] Wu, W.-N., Yuan, W.-B., Tang, N., Yang, R.-D., Yan, L. & Xu, Z.-H. (2006). *Spectrochim. Acta Part A*, **65**, 912–918. 10.1016/j.saa.2006.01.031 16914368

